# Bioremediation of Pb contaminated water using a novel *Bacillus* sp. strain MHSD_36 isolated from *Solanum nigrum*

**DOI:** 10.1371/journal.pone.0302460

**Published:** 2024-04-29

**Authors:** Pfariso Maumela, Sinomncedi Magida, Mahloro Hope Serepa-Dlamini

**Affiliations:** Faculty of Science, Department of Biotechnology and Food Technology, University of Johannesburg, Doornfontein Campus, Doornfontein, Johannesburg, South Africa; Dr Rammanohar Lohia Avadh University, INDIA

## Abstract

The Pb bioremediation mechanism of a multi-metal resistant endophytic bacteria *Bacillus* sp. strain MHSD_36, isolated from *Solanum nigrum*, was characterised. The strain tested positive for the presence of plant growth promoters such as indoleacetic acid, 1-aminocyclopropane-1-carboxylate deaminase, siderophores, and phosphate solubilization. The experimental data illustrated that exopolysaccharides and cell hydrophobicity played a role in Pb uptake. The data further showed that the cell wall biosorbed a significant amount (71%) of the total Pb (equivalent to 4 mg/L) removed from contaminated water, compared to the cell membrane (11%). As much as 11% of the Pb was recovered from the cytoplasmic fraction, demonstrating the ability of the strain to control the influx of toxic heavy metals into the cell and minimize their negative impacts. Pb biosorption was significantly influenced by the pH and the initial concentration of the toxic ions. Furthermore, the presence of siderophores and biosurfactants, when the strain was growing under Pb stress, was detected through liquid chromatography mass spectrometry. The strain demonstrated a multi-component based Pb biosorption mechanism and thus, has a great potential for application in heavy metal bioremediation.

## Introduction

Lead (Pb) is a common toxic heavy metal widely distributed in the environment because of anthropogenic activities such mining, manufacturing, transportation, and natural activities including volcanic explosions, natural weathering, as well as veld fires [1,2]. Pb is found in gasoline and used in the manufacturing of batteries, ammunition, painting, water pipes, alloys, glassware, and reaction tanks for the chemical industry [3]. The prevalence of Pb contamination in soil and water is a major health concern because the metal is hazardous at low blood levels, particularly to children [2]. The US Centres for Disease Control (CDC) and Prevention reported that Pb blood levels as low as 0.1 mg/L are toxic in children [4]. Pb can disrupt the human endocrine system in blood concentrations as low as 10 mg/L [5]. Moreover, Pb is associated with medical conditions such as cancer, anemia, cardiovascular disease, hepatitis, encephalopathy, renal failure, liver damage, and hematopoietic [3,6].

Water and soil pollution, due to the high mobility of Pb, has detrimental impacts on the aquatic and terrestrial biodiversity [7]. The increased accumulation of Pb in soil and water has an adverse impact on plant growth and development as well as the microbial diversity thereby, disrupting the well being of the ecosystem [1]. Pb pollution is also a huge problem because of the metal’s ability to enter the food chain at different trophic levels and to biomagnify at the different strata of the ecosystem [8].

Currently physical and chemical technologies such as flotation, ion exchange, sedimentation, precipitation, granulated activated carbon (GAC) adsorption and electrochemical disposition are used for Pb remediation [9,10]. However, these technologies have proven to be ineffective and unsustainable [11]. This is partly attributed to the long-term persistence of Pb in the environment because it is non-degradable [12]. Furthermore, these technologies are prone to major drawbacks such as high capital and operational costs, problems with large scale applications, low sensitivity, energy extensive and generation of secondary pollutants [13,14]. Hence, there is a need to explore and develop the potential of bioremediation as an alternative, environmentally friendly and sustainable technology for Pb remediation [13,15].

Bioremediation is widely applied in the decontamination of many industrial pollutants and for the rejuvenation of the environment [16]. Bioremediation involves the use of microorganisms or biological material for the remediation of pollutants [13,17]. Bacteria have drawn much interest in bioremediation because they are versatile and of their ease of adaptability [18]. Bacteria are capable of growth under adverse environments because they develop adaptable mechanisms [9]. Moreover, bacteria-based bioremediation is appealing because it is cost-effective and eco-friendly [5]. The effectiveness of using bacteria in bioremediation is influenced by factors such as the ability of bacteria to survive in contaminated environments [19], the rate of expression of detoxification genes, influence of abiotic factors on bacterial growth, influence of pollutants on the activity of bacteria and the mechanism of metal detoxification [20].

Bacteria have adapted mechanisms such as efflux and biosorption to survive in Pb contaminated environments [21]. The efflux mechanism is dominant in a large group of Pb resistant bacterium and involves the active transport of toxic metal ions out of the bacterial cell into the periplasm or the environment [22]. The transport of Pb out of the cell during the efflux mechanism prevents damage to intracellular biomolecules [21]. On the other hand, biosorption involves the adsorption of Pb ions on the bacterial cell surface through physical and chemical interaction [23]. The efficiency of Pb adsorption by bacterial cells is attributed to their small size and short generation time [20]. Adsorption is influenced by environmental factors such as pH, temperature, the concentration of the sorbent and metal ion [23]. Moreover, bacteria synthesize biosurfactants, extracellular polymeric substances (EPS) and siderophores that prevent, moderate, or neutralise the toxicity of heavy metals such as Pb [24,25].

The overall aim of this study was to assess the potential of the *Bacillus* sp. MHSD_36 strain, an endophyte isolated from *Solanum nigrum*, in the bioremediation of Pb contaminated water. The study interrogated the role of the cell wall and membrane components, EPS, and cell hydrophobicity as well as the impact of reaction conditions on the bioremediation of Pb.

## Materials and methodology

### Plant material collection and bacterial endophyte isolation

The plant material leaves were collected from Botlokwa, Ga-Ramatšowe, Limpopo Province, South Africa (-23.491054, 29.746048), in March 2017 from a site with sandy soil. The plant material was placed in sterile polyethylene bags and transported to the laboratory under 4°C. The identification of the plant material was carried out at the University of Johannesburg Herbarium (JRAU). A sample specimen of the plant material was deposited in JRAU with voucher specimen number Serepa-Dlamini 209 and species name *Solanum nigrum*. The remaining collected plant material was immediately processed in the laboratory. The bacterial endophytes were isolated from the leaves of *S*. *nigrum* which were sequentially washed and sterilised using distilled water for 1 min, 70% ethanol for 1.5 min, 1% sodium hypochlorite (NaOCl) for 3 min and washed in sterile distilled water three times. The water from the final wash was then plated as a negative control. The surface sterilised plant material was ground in 2 mL of saline with a sterile pestle and mortar and the resultant homogenate was then streaked onto nutrient agar (NA) plates following sterile techniques. Bacterial growth was inspected daily following incubation of the plates for 24–48 h at 28°C. The grown colonies were re-cultured thrice using the above-mentioned growth conditions in NA to get pure colonies with uniform morphology. For each bacterial endophyte, 30% (v/v) glycerol stock cultures were prepared and stored at -80°C for future use.

### Bacterial strains maintenance and growth

A 30% glycerol stock of the bacterial culture was plated on NA plates and incubated for 24 h at 28°C, for routine culture maintenance. The bacteria culture was grown on nutrient broth (NB) at 28°C, 150 rpm for 24 h.

### ACC deaminase production

The production of 1-aminocyclopropane-1-carboxylate (ACC) deaminase screening involved spot-inoculating the bacterial isolates into Dworkin and Foster (DF) minimal media supplemented with 3 mM ACC as the nitrogen source. Bacterial growth represented a positive outcome for ACC production [26].

### Indoleacetic acid (IAA) production

The production of Indole acetic acid was analyzed according to the method of Gordon and Weber [27]. A freshly grown bacterial culture was inoculated on sterile DF minimal medium supplemented with 0.5 mg/mL of tryptophan and incubated for 4 days at 30°C. Two mL Salkowski Chromogenic Agent was mixed with 10 mL of the cell suspension. The mixture was subsequently allowed to stand in the dark for 30 min at 25°C. The production of a pink colour from the reaction was a positive indicator for IAA production.

### Mineral phosphate solubilization activity

A bacterial culture was inoculated in the middle of Pikovskaya [28] agar plates, containing insoluble inorganic phosphate, and incubated at 28°C for 7 days. A halo or yellow zone around the colony was indicative of phosphate solubilization activity.

### Siderophore production

The siderophore production of the strain was determined using the Chrome Azurol S agar (CAS) method of Schwyn and Neilands [29]. The strain was inoculated in the centre of a CAS agar plate and incubated at 28°C for 3 days. An orange halo zone around the colony was evidence of siderophore production.

### Heavy metal resistance and tolerance

Heavy metal resistance and tolerance was determined using the agar dilution method. The isolated strain was inoculated onto Luria Bertani (LB) (peptone 10 g/L, yeast extract 5 g/L, NaCl 5 g/L dextrose hydrate 10 g/L and agar 30 g/L, pH 7) agar plates containing heavy metals (Zn, Pb, Cu) at a concentration of 150 mg/L, based on the minimum inhibitory concentration assay for the three heavy metals [Unpublished data]. The metal salts used to supplement the LB agar were added after autoclaving and cooling to 50°C. The plates were then incubated at 30°C for 48 h. The control plates were prepared with growth on LB media without the heavy metal salts and used to compare growth with heavy metal treated plates.

### Screening for exopolysaccharide production

The bacterial strain was tested for exopolysaccharide (EPS) production according to the method of Khalil *et al* [30]. The strain was first streaked on NA plates supplemented with sucrose 5% (w/v) and incubated at 37°C for 72 h. Nutrient broth (NB) was inoculated with a single colony of the bacterial culture and incubated at 30°C with agitation at 150 rpm for 24 h. One mL of the culture was inoculated into fresh broth and incubated for 72 h at 30°C and agitating at 150 rpm. Twenty-five mL of the cell suspension was mixed with 0.2 mL of 5 mM EDTA and NaCl or 0.2 mL dH_2_O for the control, mixed vigorously by vortexing and centrifuged at 8000 rpm for 40 min at 4°C. The cell-free supernatant was discarded, and pellet resuspended in 5 mL of deionised water with 10 mg/L Pb. The mixture was incubated at 25°C agitating at 150 rpm for 24 h and the concentration of residual Pb determined with Inductively coupled plasma optical emission spectroscopy (ICP-OES). The phenol-sulfuric acid assay was used to determine the presence of EPS.

### Cell surface hydrophobicity

Bacterial cells were harvested from the broth by centrifugation at 7000 rpm for 15 min and washed twice with 5 mL of phosphate buffered saline (PBS) pH 7. The washed cells were resuspended in 2 mL of PBS, mixed with an equal volume of chloroform and vortexed for 2 min. The aqueous and organic phases were allowed to separate for 30 min at room temperature (±25°C). The aqueous phase was separated (cell hydrophobicity components) from the organic phase and mixed with 10 mg/L of Pb to a final volume of 5 mL using PBS. The organic phase was subsequently resuspended in 5 mL PBS with 10 mg/L of Pb and incubated at 25°C and agitating at 150 rpm for 24 h. The concentration of Pb in the solution was subsequently determined with ICP-OES. The control treatment was treated with distilled water instead of chloroform.

### Subcellular fractionation

Subcellular fractionation was obtained according to the method of Kumar and Upreti [31]. The cells were grown with Pb (10 mg/L) and without Pb for 24 h and harvested by centrifugation at 7000 rpm and 4°C for 25 min. The cells were subsequently washed twice with 0.03 M Tris buffer containing 2.5 X 10^−3^ mol/L EDTA, pH 8.0, and then resuspended in the buffer. The spheroplasts was prepared by adding lysozyme to a final concentration of 200 mg/mL and incubation for 30 min at 25°C. Spheroplasts were collected by centrifugation at 3500 rpm for 15 min and resuspended in 0.03 M Tris buffer containing 3 x 10^−3^ mol/L EDTA, pH 8. The supernatant consisted of the periplasmic fluid. The spheroplasts were disrupted by two 15 seconds (s) bursts with the Vibronic Ultrasonic processor and centrifuged at 3000 rpm for 10 min to remove debris and unbroken cells. The resulting supernatant consisting of membrane and cytoplasmic fractions was centrifuged at 3500 rpm for 15 min. The pellet consisted of both outer and inner membrane envelopes. The different fractions were used for the Pb determination using ICP-OES. The solution from the assays was centrifuged at 4000 rpm for 10 min and filter sterilised with a 0.45 μm syringe filter before analysis.

### Genome extraction, Library preparation, and sequencing

Genomic DNA was extracted from solid colonies using the NucleoSpin microbial DNA extraction kit according to the manufacturer’s protocol (Macherey-Nagel, Germany). The DNA was sequenced at a commercial service provider, Biotechnology Platform, Agricultural Research Council, Onderstepoort, South Africa. Paired-end libraries (2 × 150 bp) were generated using the MGIEasy Universal DNA Library preparation kit (MGI Tech Co., China), and sequencing was performed on the MGIEasy® platform.

### Genome assembly and annotation

The genome quality control, trimming, and assembly were performed on GALAXY accessible from https://usegalaxy.org/ [32]. The FastQC (v 0.72.0) was used for quality control of the raw sequence reads followed by trimming with the Trimmomatic program (version 0.38.0) [33]. The sequence reads were *de novo* assembled using Unicycler (v 0.4.8.0) [34], and the quality was assessed with Quast (Galaxy v 5.0.2) [35]. The draft genome was annotated using the National Center for Biotechnology Information—Prokaryotic Genome Annotation Pipeline (PGAP) [36] and Rapid Annotations using Subsystems Technology [37].

### Phylogenetic analysis

The whole genome-based taxonomic analysis was performed using a free bioinformatics platform, Type Strain Genome Server (TYGS), accessible from; https://tygs.dsmz.de [38]. The pairwise comparisons among the set of genomes were performed with the Genome Blast Distance Phylogeny and accurate intergenomic distances inferred under the algorithm trimming and distance formula *d2* [39]. The average nucleotide identity (ANI) values between the strain and closely related species were calculated with Orthologous Average Nucleotide Identity Tool (OAT) software [40]. The Genome-to-Genome Distance Calculator was performed from https://ggdc.dsmz.de/ [39].

### Liquid chromatography–mass spectrometry analysis (LC-MS)

The bacterial excretome, following exposure to Pb, was analysed with a liquid chromatography-quadrupole time-of-flight tandem mass spectrometer (LC-MS-9030 q-TOF, Shimadzu Corporation, Kyoto, Japan) fitted with a Shim-pack Velox C_18_ column (100 mm x 2.1 mm with particle size of 2.7 m). The column oven temperature was maintained at 50°C. The injection volume was 5 μL, and the samples were analytically separated over a 30 min binary gradient. A constant flow rate of 0.04 mL/min was applied using a binary solvent mixture of water with 0.1% formic acid (Eluent A) and 0.1% formic acid in acetonitrile (Eluent B). The gradient technique was gradually increased from 3 to 30 min to facilitate the separation of the compounds within the samples. Eluent B was kept at 5% from 0 to 3 min, gradually increased from 5 to 40% between 3 and 5 minutes, and finally increased to 95% between 5 and 23 min. Eluent B was subsequently kept isocratic at 95% between 23 and 25 min. The gradient was returned to original conditions of 5% at 25–27 min, and re-equilibration at 5% occurred at 27–30 min. The liquid chromatographic eluents were subsequently subjected to a Quadruple Time-of-Flight high-definition mass spectrometer for analysis in positive electrospray ionization (ESI) mode with the following conditions: 400°C heat block temperature, 250°C Desolvation Line (DL) temperature, 42°C flight tube temperature, and 3 L/min nebulization and dry gas flow. The data was acquired using the Data-dependent acquisition (DDA) mode, which simultaneously generated MS1 and MS2 data for all ions within a mass-to-charge ratio (m/z) range of 100–1500 (precursor m/z isolation window) and an intensity threshold above 5000. The MS2 Experiments were conducted out utilizing argon gas as the collision gas and a collision energy of 35 eV with a spread of 5 and sodium iodide (NaI) as a calibration solution to monitor high mass precision.

### Experimental design: Factors impacting the biosorption of Pb

A five-coded level central composite design (CCD) was used to determine the impact of the initial metal-ion concentration, pH of the reaction environment and concentration of the biosorbent, on Pb removal. The experiment had 20 runs including 6 center points. The factors were used with the following range, Pb concentration (7.4, 50, 112.5, 175, 217.6 mg/L), pH (2.6, 4.0, 6.0, 8.0, 9.4), and biosorbent concentration (0.66, 1.0, 1.5, 2.0, 2.34 g/L). The general formula for the response is shown in the equation below:

$$y_{i}=\beta_{0}+\sum_{i=1}^{n}\beta_{i}x_{i}+\sum_{i=1}^{n}\beta_{ii}x_{i}^{2}+\sum_{i<j}\beta_{ij}x_{i}x_{j}+\epsilon$$ (1)

where y_i_ is the i^th^ response variable, x_i_ is the i^th^ input parameter, n is the number of input parameters and β_0_, β_i_, β_ii_, β_ij_ are the fixed response, linear, quadratic, and cross products coefficients, respectively. Design-Expert (Design–Expert, Stat-Ease Inc. Minneapolis, MN, USA) was used for the experimental design and statistical analysis at 5% level of significance.

### Statistical analysis

All the experiments were performed in triplicates and results were presented in the form of the mean ± SD. The standard deviations were represented in charts as error bars. The significant difference was determined by the ANOVA in Microsoft Excel 365. The ANOVA was performed at 5% level of significance.

## Results

### Strain identification

**Fig 1:**
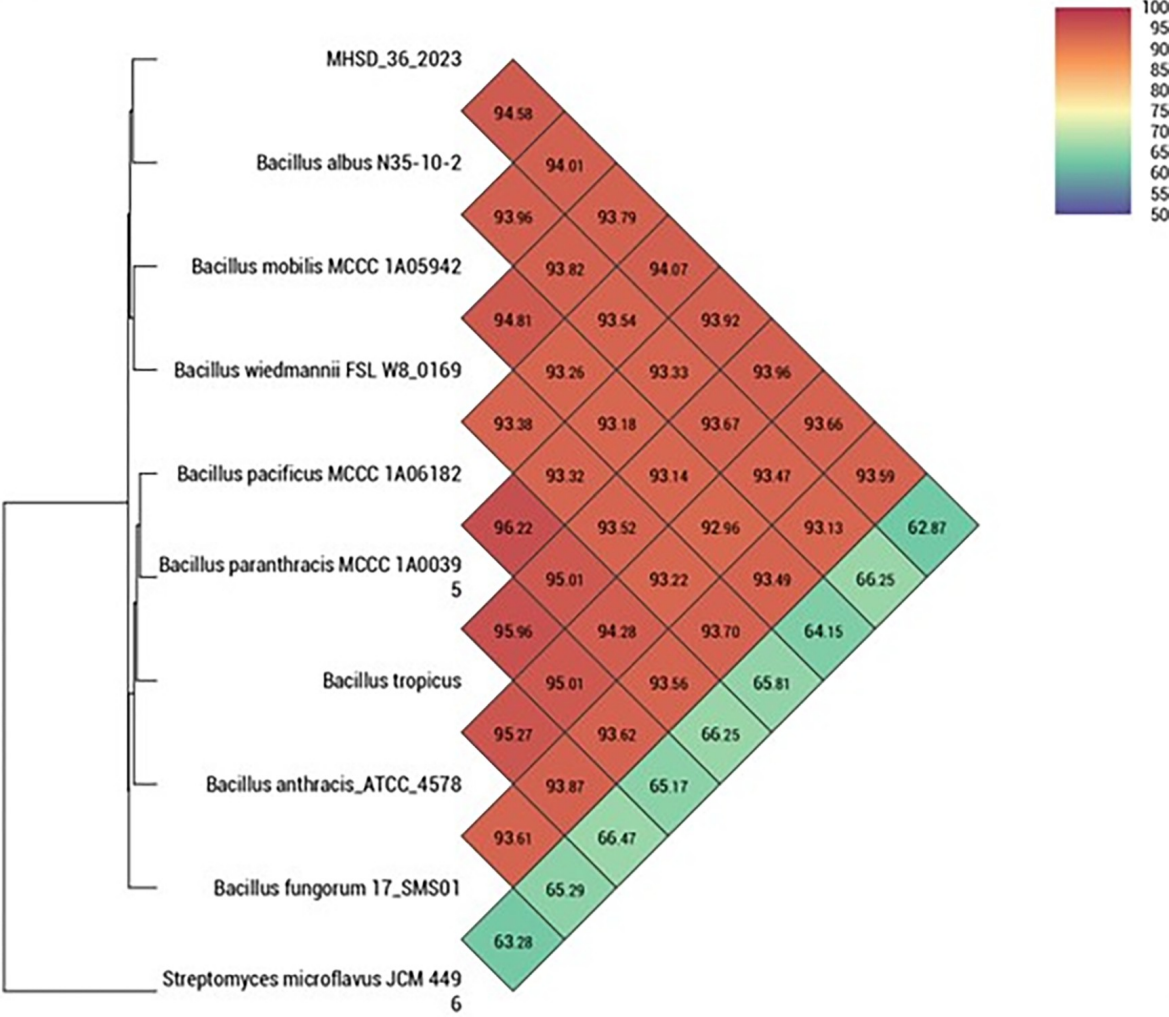
Heatmap for the ANI analysis of strain MHSD_36 with closely related species.

The genome sequence of *Bacillus* sp. strain MHSD_36 (accession number JAVBIS000000000) was 5139851 bp, with a genomic DNA G+C content of 35.3%, and N_50_ of 782215 bp. The DNA G+C content and genome size of strain MHSD_36 was comparable to other *Bacillus* strains (Table 1). A total of 5375 of genes of which 5118 were protein-coding genes, 171 pseudogenes and 86 RNA genes were identified through PGAP. Furthermore, strain MHSD_36 recorded digital DNA–DNA hybridization (dDDH) values above the recommended cutoff point of 70% for species delineation with *Bacillus* strains (Table 1). Strain MHSD_36 was closely related to *Bacillus tropicus* N24 with a dDDH value of 79%, and G+C content difference of 0.11, nonetheless, this was lower than subspecies delineation threshold of dDDH >79–80%. The ANI analysis however, illustrated that strain MHSD_36 was closely related to *Bacillus albus* N35-10-2 with an ANI value of 94.6% (Fig 1). The GGDC data (S1 Table) showed that MHSD_36, based on the recommended Formula 2, belonged to a *Bacillus wiedmannii* strain. However, the strain was distinct from the reference strain *Bacillus wiedmannii* strain FSL W8-0169 (LOBC01000053) with a G+C content difference of 15.76.

**Table 1:** The global alignment and pairwise comparison of *Bacillus* sp. MHSD_36 with related species.

Subject strain	dDDH (d0, in %)	C.I. (d0, in %)	dDDH (d4, in %)	C.I. (d4, in %)	dDDH (d6, in %)	C.I. (d6, in %)	G+C Content	G+C content difference (in %)
*Bacillus tropicus* N24	77.3	[73.3–80.8]	53.6	[50.9–56.3]	74.5	[71.0–77.7]	35.44	0.11
*Bacillus paranthracis* MCCC 1A00395	75	[71.0–78.6]	54.2	[51.5–56.9]	72.8	[69.3–76.0]	35.46	0.13
*Bacillus pacificus* MCCC 1A06182	74.6	[70.6–78.2]	55.2	[52.5–57.9]	72.8	[69.3–76.0]	35.44	0.11
*Bacillus wiedmannii* FSL W8-0169	74.4	[70.4–78.0]	53.3	[50.6–55.9]	72	[68.6–75.3]	35.45	0.12
*Bacillus cereus* ATCC 14579	73.4	[69.4–77.0]	43.8	[41.2–46.3]	67.8	[64.4–71.1]	35.34	0.03
*Bacillus anthracis* ATCC 14578	72.8	[68.8–76.4]	52.5	[49.8–55.1]	70.4	[67.0–73.7]	35.38	0.07
*Bacillus albus* N35-10-2	67.8	[63.9–71.4]	58.2	[55.4–61.0]	67.8	[64.3–71.0]	35.69	0.38
*Bacillus luti* MCCC 1A00359	67.7	[63.8–71.3]	43.7	[41.1–46.2]	63.2	[59.9–66.4]	35.44	0.13
*Bacillus mobilis* MCCC 1A05942	67.1	[63.2–70.7]	53.6	[50.9–56.3]	66	[62.6–69.2]	35.35	0.04
*Bacillus toyonensis* NCIMB 14858	67.1	[63.2–70.7]	42.3	[39.8–44.9]	62.3	[59.0–65.5]	35.55	0.24
*Bacillus thuringiensis* ATCC 10792	61.4	[57.7–64.9]	43.5	[41.0–46.1]	58.1	[54.9–61.2]	35.8	0.49
*Bacillus fungorum* 17-SMS-01	60	[56.3–63.5]	52.7	[50.0–55.3]	59.6	[56.3–62.8]	35.65	0.34
*Bacillus paramycoides* NH24A2	55.8	[52.2–59.3]	36.8	[34.3–39.3]	51.2	[48.2–54.3]	33.43	0.12
*Streptomyces microflavus* JCM 4496	12.5	[9.8–15.8]	63.4	[60.5–66.2]	12.9	[10.6–15.7]	71.23	35.92

### Heavy metal tolerance

**Fig 2:**
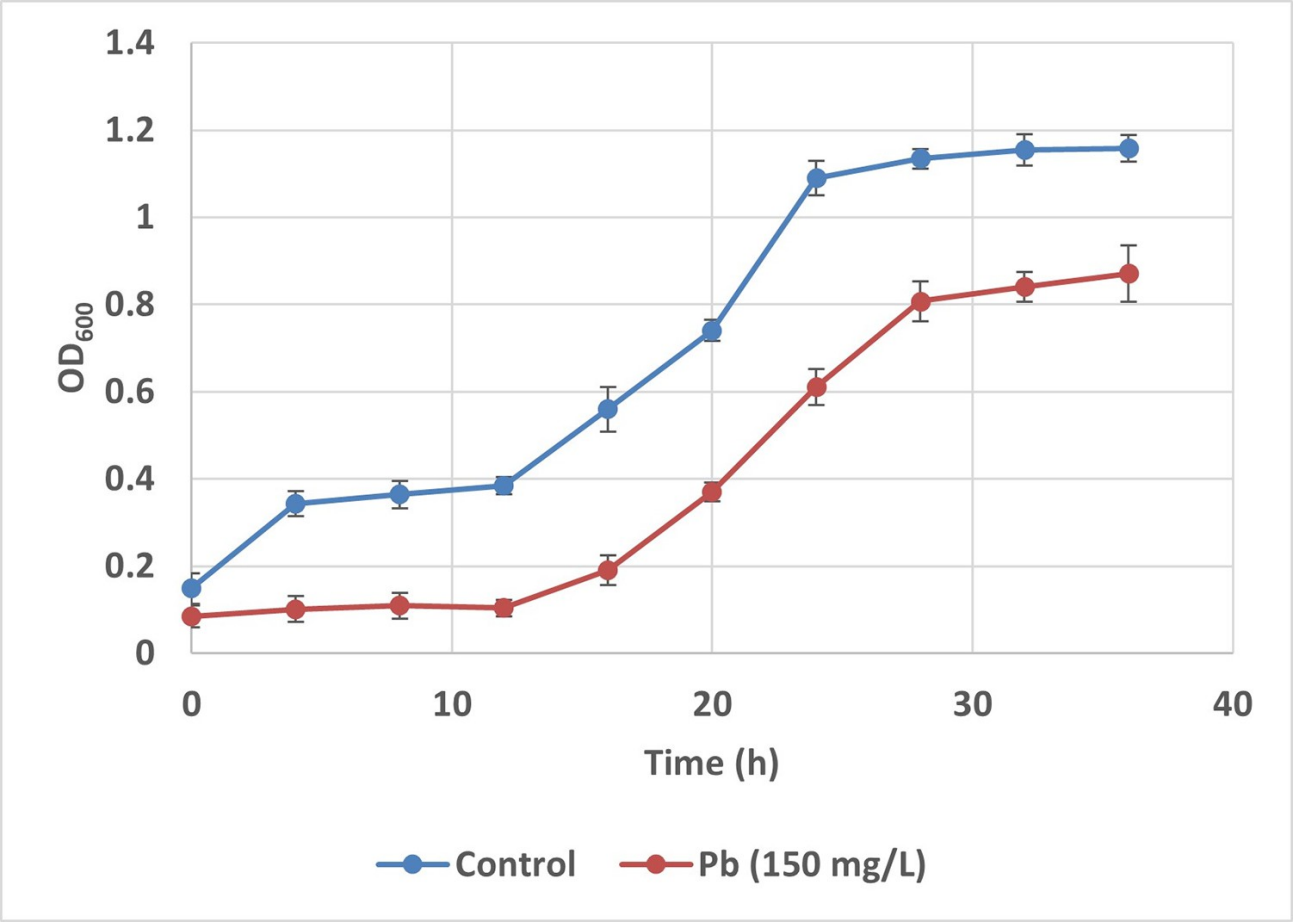
Strain MHSD_36 growth in the presence of Pb. Data represents mean values ± standard deviation of the three replicates.

Strain MHSD_36 showed metal tolerance to Zn, Pb, and Cu (S2 Table) at a minimum concentration of 150 mg/L. Fig 2 is an illustration of the growth of strain MHSD_36 under Pb stress and normal conditions, without toxic heavy metal ions. The growth curve shows a longer lag phase when the strain is exposed to heavy metal stress. The duration of the lag was approximately 12 h, compared to approximately 4 h under normal conditions (Fig 2). Furthermore, the strain achieved an OD_600_ of approximately 0.8 after 28 h, compared to 1.1 under normal conditions, thereby showing a good ability to detoxify Pb and bioremediation capability. Interestingly, the genome annotation of strain MHSD_36, identified genes involved in heavy metal resistance (Table 2). The genome annotation identified the *czcR* and *czcD* genes which are responsible for the induction of cobalt-zinc-cadmium resistance. The gene encoding for the protein domain of the zinc/cadmium/mercury/lead-transporting ATPase (Table 2) was also identified.

**Table 2:** A summary of *Bacillus* sp. strain MHSD_36 genes important in toxic heavy metal bioremediation.

Role	*Gene*	Gene Product
Exopolysaccharide synthesis	*EpsF*	Exopolysaccharide biosynthesis glycosyltransferase
	*EpsA*	Exopolysaccharide biosynthesis transcriptional activator
Toxic heavy metal resistance	*zntA*	zinc/cadmium/mercury/lead-transporting
	*czcD*	Cobalt-zinc-cadmium resistance protein
	*czcR*	Cobalt-zinc-cadmium resistance protein
Siderophore synthesis	*Ents*	Enterobactin exporter
	*dhbF*	Bacillibactin synthetase component F
	*dhbC*	Isochorismate synthase
	*dhbE*	2,3-dihydroxybenzoate-AMP ligase
	*yuiI*	Trilactone hydrolase
Fe uptake	*PB_PBP*	Petrobactin ABC transporter, periplasmic binding protein
	*PB_PPI*	Petrobactin ABC transporter, permease protein I
	*PB_PPII*	Petrobactin ABC transporter, permease protein II
	*PB_ABP*	Petrobactin ABC transporter, ATP-binding protein
Plant growth promoters	*Sap*	secreted alkaline phosphatase
	*phoP*	Alkaline phosphatase synthesis transcriptional regulatory protein
	*Ppk*	Polyphosphate kinase
	*Epp*	Exopolyphosphatase
	*Iah*	The Indole-3-acetamide hydrolase gene

### Plant growth promoting factors (PGPFs)

Strain MHSD_36 tested positive for the synthesis of plant growth promoters indoleacetic acid (IAA), 1-aminocyclopropane-1-carboxylate (ACC) deaminase, siderophores, and phosphate solubilization (S3 Table). The *in vivo* assay data was consistent with the genome annotation analysis which identified the presence of genes coding for the enzymes, isochorismatase (*dhb*B), isochorismate synthase (*dhb*C) and bacillibactin synthetase component F (*dhbF*), involved in the biosynthesis of bacillibactin siderophore (Table 2). The indole-3-acetamide hydrolase gene (*iah*), responsible for the conversion of indole-3-acetamide (IAM) to the phytohormone IAA was also identified. The genes coding for the siderophore, petrobactin, and assisted-ABC transporter proteins (PB_PBP, PB_ABP, PB_PPI and PB_PPII) were also identified from the genome of strain MHSD_36 (Table 2).

### Effects of EPS on Pb accumulation

**Fig 3:**
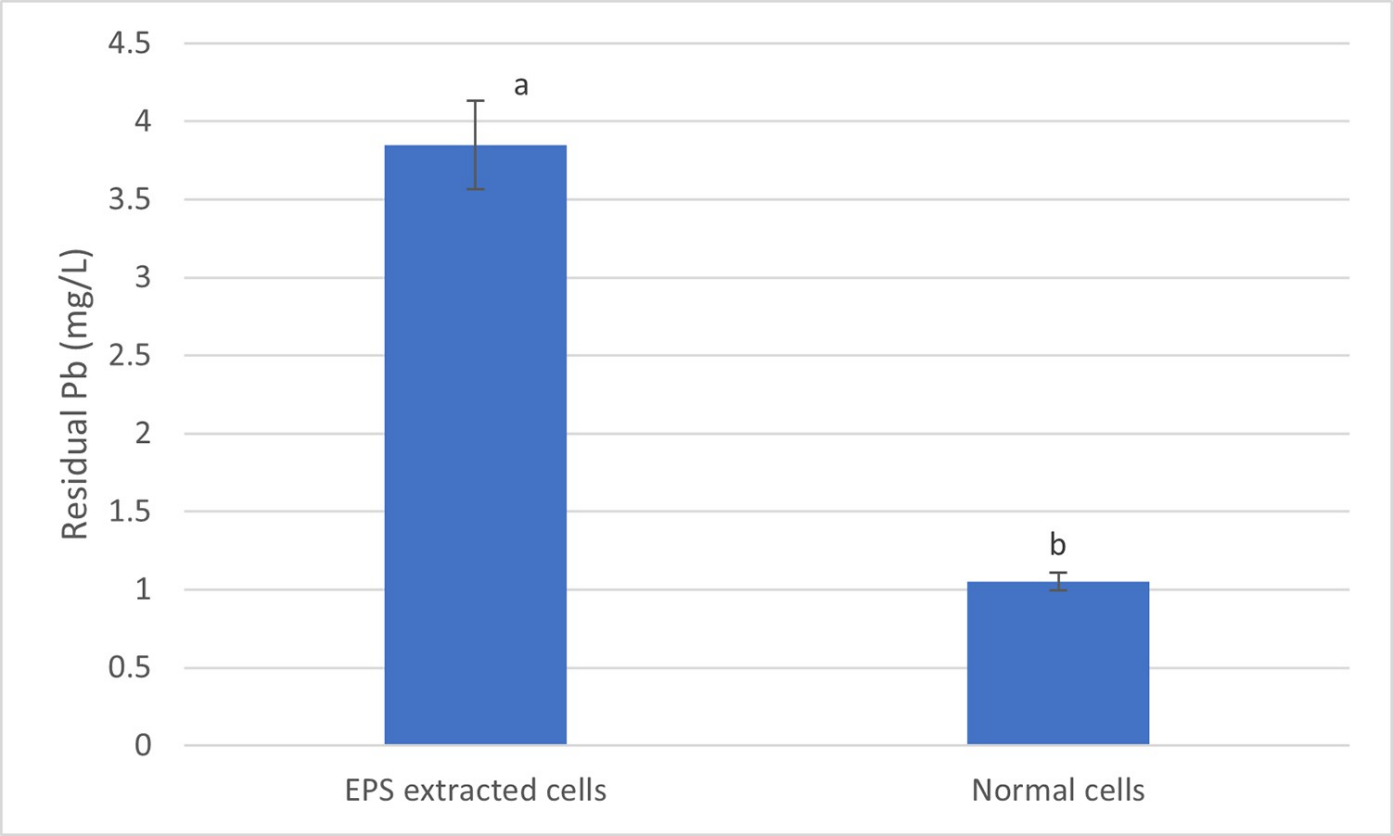
Comparison of residual Pb in contaminated water treated with normal and EPS extracted cells. The data represents mean values ± standard deviation of the three replicates. The different letters represent significant differences (p < 0.05).

The role of the EPS in Pb bioremediation, from contaminated water, was examined by comparing the residual Pb concentration between samples treated with normal cells and EPS extracted cells. The concentration of residual Pb after a 24 h exposure was higher in the latter, at approximately 4 mg/L and almost double compared to the treatment with normal cells (Fig 3). The difference between the concentration of the residual Pb from the normal cell treatment and the EPS extracted cells (Fig 3) shows that approximately 50% of Pb removed from the contaminated water could be attributed to EPS.

### The impact of cell surface hydrophobicity in Pb accumulation

**Fig 4:**
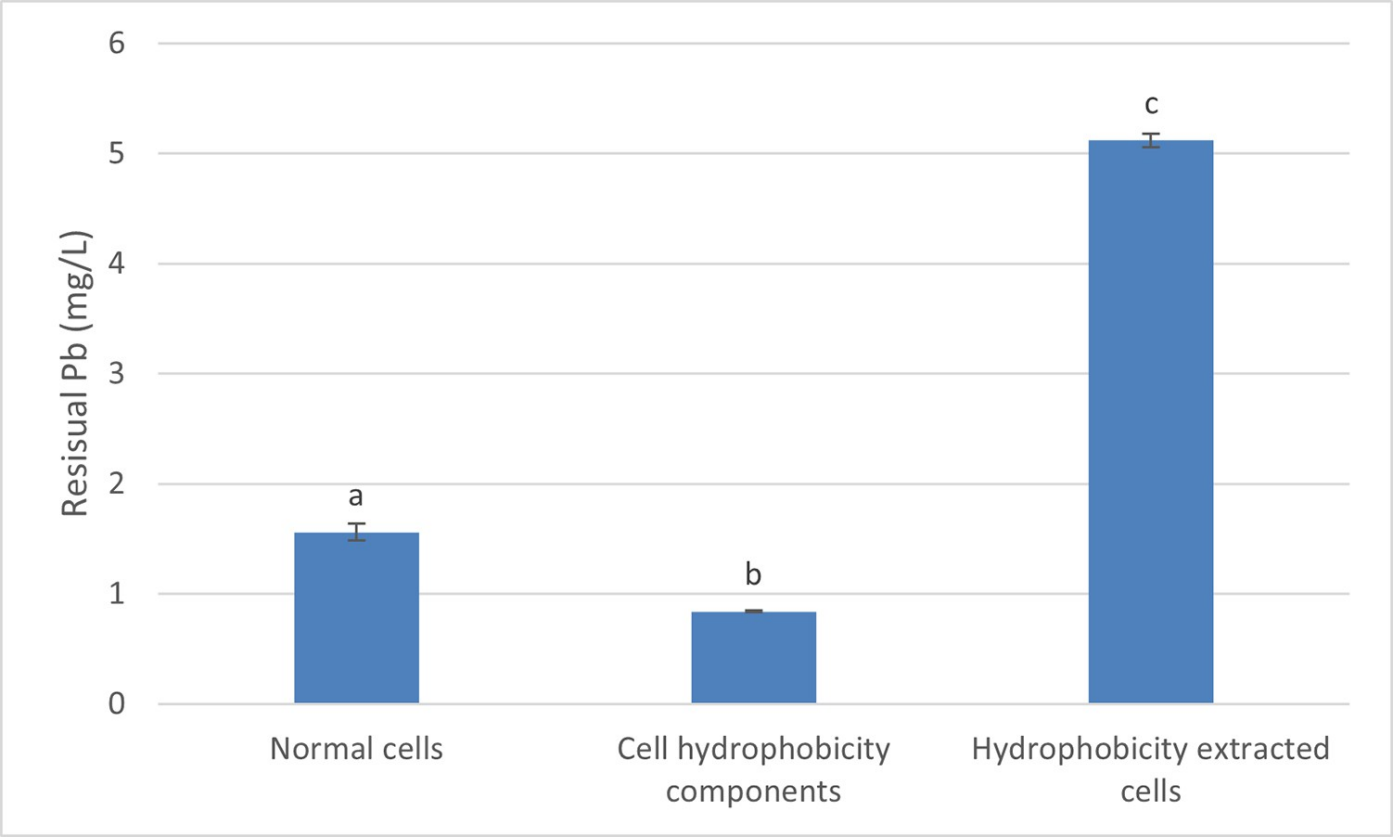
Comparison of the residual Pb following the incubation of contaminated water with normal cell, cell hydrophobicity extracted cells and cell hydrophobicity extract. Data represents mean values ± standard deviation of the three replicates. The different letters represent significant differences (p < 0.05).

The experimental data summarised in Fig 4 shows that cell hydrophobicity plays a role in the Pb bioremediation by the *Bacillus* sp. MHSD_36. The concentration of the residual Pb in the treated cells was twice as much (5 mg/L) as that of normal cell treatment (Fig 4) thereby illustrating that cell surface hydrophobicity plays a role in Pb removal form contaminated water. Treating the Pb contaminated water with an extract of the cell surface hydrophobicity components also resulted in a lower residual concentration (1 mg/L) of the Pb compared to the treated and normal cells (Fig 4).

### The relevance of cell wall and cell membrane in the Pb accumulation by *Bacillus*

**Fig 5:**
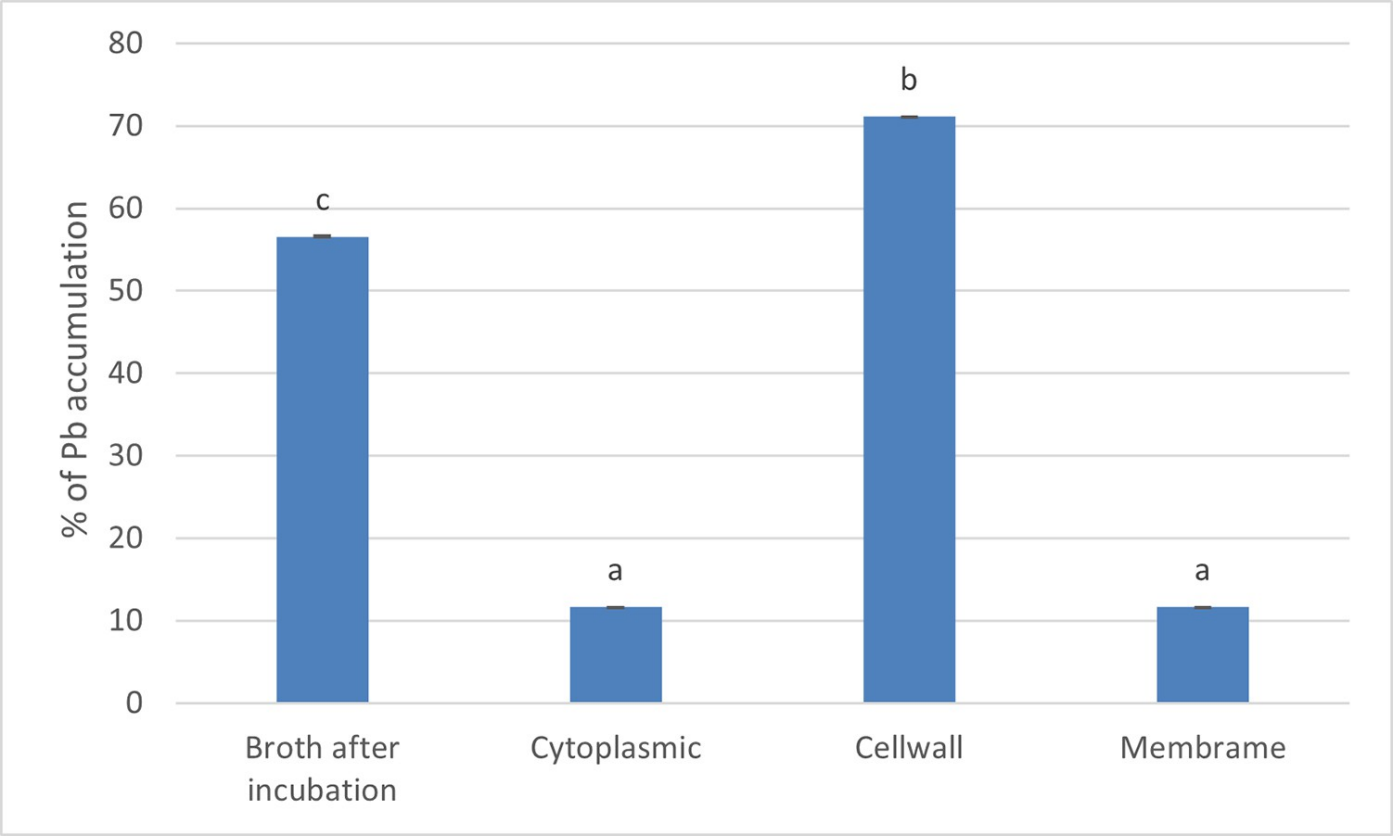
Biosorption and distribution of Pb by *Bacillus* sp. MSHD_36 strain. The percentage (%) recovery of Pb from the cell wall, cell membrane and cytoplasm are based on the total amount (%) of Pb removed from the contaminated water. The data is represented as mean values ± standard deviation of the three replicates. The different letters represent significant differences (p < 0.05).

Fig 5 is a summary of the experimental data to determine the role of the cell wall and cell membrane on Pb accumulation by strain MHSD_36. The data illustrates that 56% of Pb was removed from the contaminated water. The results show that 71% of the removed Pb was recovered from the cell wall. On the other hand, the cell membrane and cytoplasmic fraction each accounted for approximately 11%. The percentage of heavy metals internalisation was in the range of 2–12 compared to 11–71% adsorbed on cell surfaces. It is interesting to note that only 93% of the removed Pb was traced to the cell wall, cell membrane and cytoplasmic fractions.

### Secondary metabolites identified from strain MHSD_36 cultured under Pb stress

The presence of siderophores, biosurfactants and stabilising agents was detected following the exposure of strain MHSD_36 to Pb (Table 3). The siderophore corneybactin, a cyclic depsipeptides, was identified from the excretome of strain MHSD_36 (Table 3). D3 (ester) was also synthesized by strain MHSD_36 under Pb stress (Table 3). The biosurfactants, empigen BR and tridecylhexaethoxylate (Table 3), were detected from the *Bacillus* sp. MHSD_36 strain. The surfactants belong to alpha amino acid and dialkyl ethers, respectively.

**Table 3:** Secondary metabolites identified from the excretome of strain MHSD_36 following exposure to Pb stress.

Precursor (m/z)	RT	Nature of compound	Compound	Biological activity
883.27	10.09	Cyclic depsipeptides	Corneybactin	Iron acquistion/siderophores
277.13	10.5	Fatty acid esters	Eudraflex	Plasticiser
597.16	11.0	Flavanoid	Okanin	Anti-HIV/antiviral
219.18	14.19	Sesquiterpenoids	Buddledin D	Anticancer
343.3	14.97	Alpha amino acid	Empigen BR	Surfactant
181.05	16.38	Alpha-keto acids	D3 (ester)	Stabilising agents & plasticiser
280.26	16.75	Fatty amide	Farnesylacetone	Anticancer
254.25	18.7	fatty amide	Palmitoleamide	Anticancer
379.24	19.11	Cinnamic acid esters	Octocrylene	Sunscreen
291.2	19.35	Cinnamic acid esters	Escalol	Sunscreen
397.38	19.42	Delta-5-steroids	Stigmastan-3,5-diene	Antituberculosis
280.26	19.55	Fatty amide	Limoleamide	Anticancer
482.41	19.78	Dialkyl ethers	Tridecylhexaethoxylate	Surfactant
311.17	19.8	Retro-dihydrochalcones	Avobenzona	Sunscreen
711.43	23.59	Glycosyldiacylglycerols	Aspafilioside A	Anticancer
631.41	25.38	Terpene glycosides	kurilensoside f	Antimicrobial
547.4	25.91	Benzoic acid esters	hatcol 200	Plasticiser

### Factors affecting biosorption of Pb by *Bacillu*s strain MHSD_36

Bacterial biomass is a highly efficient and cost-effective heavy metal biosorbent. Moreover, strain MHSD_36 has been shown to be a potential biosorbent for the bioremediation of Pb from contaminated water (Fig 5). Heavy metal biosorption is influenced by factors such as pH, temperature, contact time, initial metal ion concentration, and the dosage of biosorbent. Therefore, further investigations were conducted to establish the impacts of pH, the concentration of heavy metal, as well as the dosage of the biosorbent on the biosorption efficiency of strain MHSD_36.

A central composite design (CCD) was applied to determine the individual and combined effects of the three parameters on the biosorption of Pb (Table 4). The experimental data was used to develop regression models for the Pb removal. The analysis of variance (ANOVA) was used to determine the significant factors affecting the Pb removal (Table 5). The adjusted coefficient of determination (adjusted R^2^) for the Pb removal was 0.8336 (Table 5). Thus, 83% of the variation in the Pb removal could be explained by a second-order polynomial model in relation to pH, initial metal ion concentration, and the dosage of biosorbent.

**Table 4:** CCD design with the actual experimental values for Pb biosorption.

Std	Run	Pb (A)	Biosorbent (B)	pH (C)	Pb removal
		mg/L	g/L		(%)
5	1	50	1	8	71.38
6	2	175	1	8	36.58
19	3	112.5	1.5	6	27.06
12	4	112.5	2.3	6	26.84
14	5	112.5	1.5	9.3	9.91
4	6	175	2	4	3.33
17	7	112.5	1.5	6	26.2
18	8	112.5	1.5	6	26.9
2	9	175	1	4	8.45
9	10	7.3	1.5	6	81.57
13	11	112.5	1.5	2.6	8.46
20	12	112.5	1.5	6	28.75
8	13	175	2	8	7.01
10	14	217.6	1.5	6	6.45
1	15	50	1	4	3.63
3	16	50	2	4	56.5
11	17	112.5	0.6	6	20.63
15	18	112.5	1.5	6	23.71
7	19	50	2	8	72.75
16	20	112.5	1.5	6	25.98

**Table 5:** Analysis of variance for the CCD model for Pb biosorption using strain MHSD_36 and coefficients for the predictive model.

		Pb removal (%)		
Source	d.f.	Co-efficient	Sum of squares	p-value
**Linear**				
Pb concentration (A)	1	-20.15	5546.63	<0.0001
Biosorbent concentration (B)	1	2.20	65.87	0.4259
pH (C)	1	8.66	1023.86	0.0084
**Quadratic**				
A^2^	1	7.66	845.94	0.0139
B^2^	1	0.4933	3.51	0.8520
C^2^	1	-4.65	311.73	0.1011
**Interaction**				
AC	1	-6.52	340.47	0.0885
BC	1	-9.49	721.05	0.0206
AB	1	-11.12	988.57	0.0092
**Regression**	19		10915.48	
**Residual error**	10		956.11	
**Lack of Fit**	5		942.46	< .0001
**Pure Error**	5		13.65	
**Model**	9		9959.37	0.0003
**R** ^**2**^		0.9124		
**Adjusted R** ^**2**^		0.8336		

**Fig 6:**
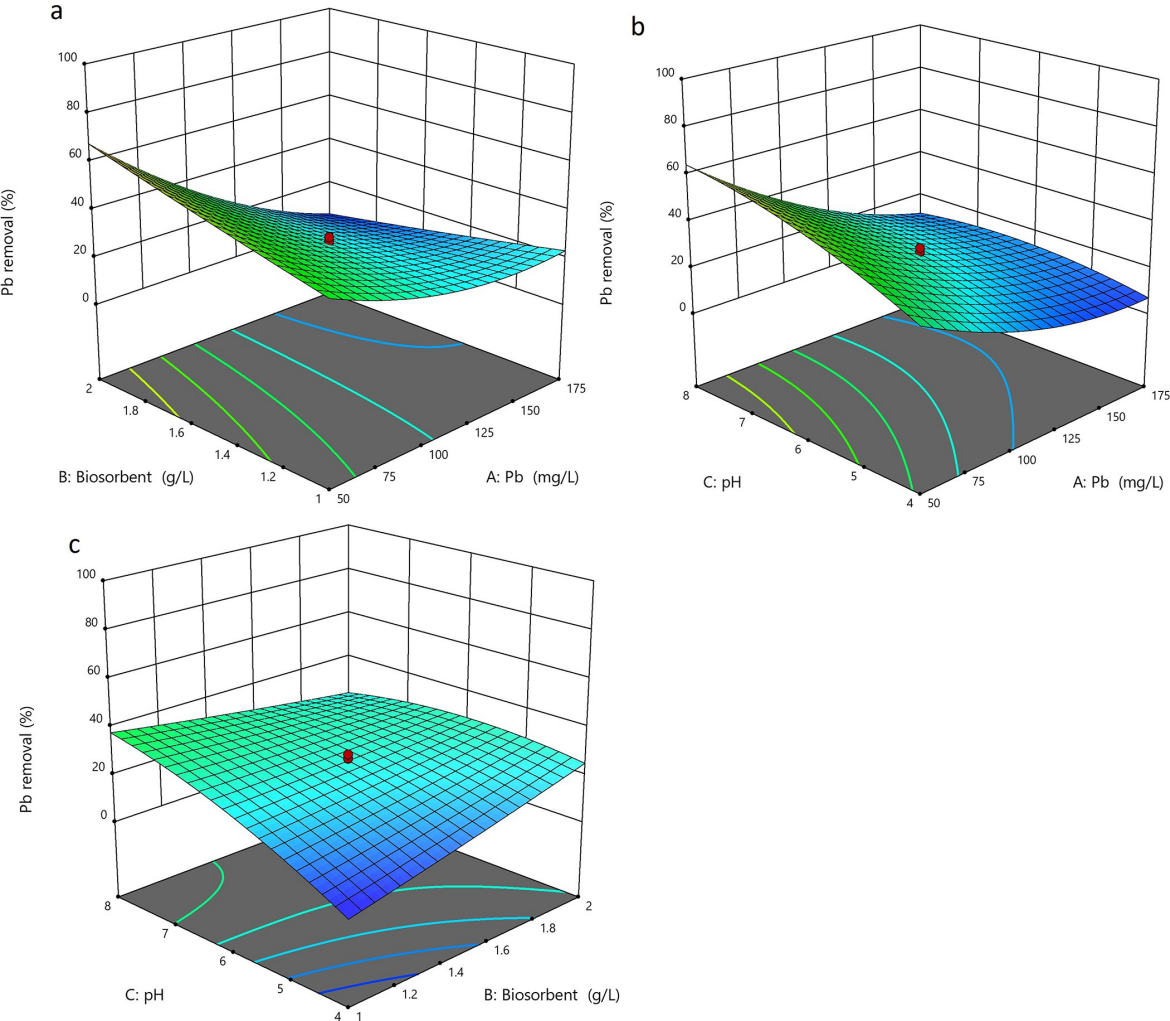
Response surface plot showing the interactive effects of the initial ion concentration (A), biosorbent concentration (B) and pH (C) on the removal of Pb.

The actual Pb removal was in the range of 8–82% (Fig 6) with a predicted maximum of 69.57% at pH, initial metal ion concentration, and the dosage of biosorbent of 7.2, 50 mg/L and 2 g/L, respectively (S1 Fig).

The initial concentration of the ions had a negative effect on the Pb removal (Fig 6A and 6B, Table 5). The interactive term of the initial ion concentration and the biosorbent concentration had a significant impact on the Pb removal (Table 5). The ANOVA (Table 5) and surface plot (Fig 6A) show that the interactive term (AB) had a negative impact on Pb removal, with lower efficiencies recorded with a simultaneous increase in both initial ion and biosorbent concentration. Pb removal increased as the pH increased from 4, acidic conditions, and reached a peak as the conditions changed towards alkane pH (Fig 6B and 6C). The negative sign on the quadratic term of pH (Table 5), also confirms that the Pb removal peaks as the pH changes towards alkaline conditions (Fig 6C). The mathematical relationship between Pb removal and the reaction is shown in Eq 2 below which summarises the significant factors;

$$y=26.21-20.15A+22B+8.66B-11AB-9BC+{7.66A}^{2}$$ 2

where *y* is the % Pb removal, *A* is the initial ion concentration, *B* is the biosorbent concentration and *C* is the pH. The regression coefficients from Eq 2 show that the initial ion concentration had the most significant impact (p < 0.0001), followed by pH and biosorbent concentrations (Table 5). The positive sign on the quadratic term of the initial ion concentration (A) illustrates a second order relationship between the Pb removal and initial ion concentration.

## Discussion

Bioremediation is a cost-effective technology that enables the exploitation of microorganisms for the removal of toxic environmental contaminants such as hydrocarbons and heavy metals [17]. The bioremediation potential of an endophytic bacteria was evaluated using *in silico* analysis and *in vitro* assays. Although the ANI analysis illustrated that strain MHSD_36 was closely related to *Bacillus albus* N35-10-2, the ANI value was below the species boundary value > 95–96% of the ANI analysis [40]. Moreover, the dDDH and ANI data were incongruent, and consequently the Genome-to-Genome Distance Calculator [39] was applied for further analysis and species delineation. Thus, based on the analysis of the dDDH, ANI and GGDC data, the endophyte MHSD_36 is a potential novel *Bacillus* sp. strain. The strain was capable of growth under Pb conditions of 150 mg/L and achieving high biomass yields, despite the longer lag phase (Fig 2).

Bacteria are widely used in bioremediation due to their ability to thrive in toxic environments and degrade or modify toxic compounds including heavy metals [19,20]. Bacteria use biosorption as the main mechanism for metal bioremediation [20,23]. Bacterial endophytes are potential candidates for bioremediation due to their symbiotic relationship with plant host which grow under adverse conditions [41,42]. Consequently, endophytic bacteria, including *Bacillus* spp., have been reported to be tolerant to toxic heavy metals such as Pb, Zn and Cd [22]. Behera *et al* [43] reported that the ATPase transporter plays a significant role in the bioremediation of heavy metal, through the efflux of toxic heavy metal ions.

Furthermore, endophytic bacteria synthesize plant growth promoting factors (S3 Table), which forms an important part of the endophytic relationship with their host plants [41,42]. PGPFs play a role in heavy metal bioremediation in addition to their role in enhancing plant growth and development for their host [24]. IAA is a plant growth promoter, which affects cell division and stimulates plant cell elongation in plants even under stress [44]. ACC is a PGPF important in the reduction of ethylene, a stress generating hydrocarbon, which subsequently promotes plant growth. Siderophores are an important group of PGPFs and are iron chelating proteins that assist plants to overcome metal stress and iron-deficiency [24]. The siderophore petrobactin (Table 2) is important for iron acquisition through membrane-associated substrate-binding proteins and ABC transporters, and therefore important for the plant host growth under iron limitation [45]. Siderophores are also capable of forming stable complex with other heavy metals such as cadmium, Pb, Zn, and Cu [46]. The presence of both IAA and siderophores has been reported to promote growth of plants under cadmium polluted soils [47], this thereby makes the bacterial endophyte, *Bacillus* sp. MHSD_36, a potential candidate for the simultaneous bioremediation and rehabilitation of contaminated soils for agricultural use.

Bacterial endophytes also synthesize and secrete EPS which are capable of binding and forming metal complexes with heavy metals ions thereby neutralising their toxic effects [25]. Moreover, EPS are also integral for biofilm formation which promotes heavy metal ions biosorption and bio-mineralisation [48]. Song *et al* [48] reported that the high level of Pb biosorption in *A*. *pullalans* compared to *S*. *cerevisiae* was attributed to the existence of EPS in the former. It has also been demonstrated that the amount of Pb biosorbed by *A*. *pullalans* increases as the concentration of EPS increases [25]. Martins *et al* [49] also reported that the treatment of Cd, Zn and Cu contaminated water with EPS resulted in 98, 53 and 51%, respectively, removal of the heavy metals. Mohite *et al* [50] reported an increase in the production of EPS when *Pantoea agglomerans* was growing in the presence of heavy metals such as Cr, Cu, Ag, Cd and Pb. Thus, the enhanced capacity of bacteria to extract Pb from polluted water was linked to the capability of EPS to adhere to heavy metal ions.

Cell surface hydrophobicity is an important attribute for bacterial survival in toxic environments and the detoxification of toxic compounds [51]. Bacteria generally increase their cell surface hydrophobicity as a defense against environmental stress, and it has been reported that a correlation between microorganisms’ hydrophobicity and the bioremediation potentially exist. The presence of functional groups, such as acetamido, sulfhydral, carboxyl, phosphodiester, phosphate, and hydroxyl on bacterial cell surfaces is an important feature of cell hydrophobicity due to their ability to adsorb to toxic heavy metals, because of differences in ion charges, and mask their toxicity through precipitation, complexation, or ion exchange [23,24]. Asri *et al* [52], reported that bacterial strains, such as *Leucobacter* sp., *Enterococcus* sp., and *Cellulosimicrobium* sp. which demonstrated higher hydrophobic characteristics had better Cr removal potential and biosorption capacity of Cr anions. Shen *et al*., (2018) showed that surface cell hydrophobicity induced heavy metal immobilisation enhanced the Cd removal efficiency of *Chlorella* sp., achieving a maximum removal efficiency of 92%. In addition, cell surface hydrophobicity modulates biofilm formation and bacterial adhesion to surfaces which play a significant role in heavy metal biosorption and detoxification [25].

The bacterial cell wall and membrane are an important barrier between the bacterial cell and the environment. The membrane plays an important role in transport which is an integral part of protecting the cell against toxic compounds from the environment [53]. Bai *et al* [54] also demonstrated that a significant amount of the metal uptake was attributed to the cell wall. Moreover, bacterial cells use energy dependent membrane efflux pumps to remove excessive toxic heavy metal ions into the periplasm and mitigate against cell cytotoxicity [55], thus promoting continued biomass growth and heavy metal biosorption. This consequently enhances the bioremediation efficiency of strain MHSD_36 because the experimental data has demonstrated that biosorption is the primary heavy metal removal method. Moreover, biosorption is an energy independent process compared to the energy dependent membrane efflux pumps, thereby making the former a preferable method of metal bioremediation [54,55] Guo *et al* [53] reported a significantly lower Pb accumulation (5.5%) from the cytoplasm of a *Bacillus* sp., compared to Pb biosorbed to the cellwall. Martin *et al* [48] also observed low levels of heavy metal (Cd, Zn and Cu) internalisation by *Paenibacillus polymyxa*. Thereby, bacterial cells use biosorption, instead of bioaccumulation, as the primary Pb bioremediation mechanism.

The bacterial cell wall is composed of lipids, proteins as well as polysaccharides which have an abundance of metal binding groups such as phosphates, sulphates, amino and carboxyl groups capable of binding to metal ions through ion exchange and complexation [56]. The metal binding groups contribute to metabolism independent biosorption of heavy metals during bioremediation. The pH has been reported to be one of the most important environmental factors in Pb biosorption because it modifies the chemical properties of metal ions and affects the ionization state of the bacterial cell wall functional groups [57]. Bacterial cell walls are composed of carboxyl functional groups which have been reported to effectively bind to metal cations in the pH range of 3–6 [58,59]. Moreover, higher biosorbent dosages have been shown to increase Pb removal if the microbial concentration is not too high to result in biomass aggregation and consequent decrease in the surface area [60]. The bacterial growth conditions are important to support growth that will result in sufficient surface area for optimal Pb adsorption on the cell surface. Moreover, conditions such as the pH should be sufficient to influence metal complexation and ion exchange [56] during the binding of the metal ions (Pb) to the charged functional groups on the cell surface.

Bacteria synthesize biosurfactants and siderophores that precipitate heavy metals or neutralise the toxicity of heavy metals such as Pb [48,61]. Kunkle and Schmitt [62] reported that *Corynebacterium diphtheriae* with mutant ciuE deletion was defective of corneybactin and consequently iron uptake and growth in low iron conditions. D3 (ester) is a biosurfactant belonging to alpha-keto acids which have been identified as novel siderophores with growth promotion characteristics and a role in iron acquisition [63]. Biosurfactants are capable of heavy metal complexation, emulsification, mobilisation, and solubilization [64]. Thereby the synthesis of EPS, together with biosurfactants and siderophores potentially contributes to the bioremediation of the Pb from the wastewater by *Bacillus* sp.

In conclusion, the strain MHSD_36 demonstrated a potential for application in the bioremediation of Pb contaminated water. The potential rests in the multi-component based Pb biosorption by the cell wall. The study demonstrated the role of the EPS and cell hydrophobicity in the removal of Pb from the contaminated water. The efficiency of the Pb removal can be enhanced by the siderophores produced by the strain. The cell wall was capable of Pb biosorption, and this constituted a significant amount of the Pb removed from the contaminated water. Moreover, biosorption of Pb by strain MHSD_36 was significantly influenced by the pH of the reaction environment and the initial concentration of the toxic ions. The use of metabolism independent mechanisms for Pb bioremediation by the *Bacillus* sp. strain MHSD_36 presents a potential for the application of the strain in heavy metal bioremediation from contaminated water with limited nutrients.

## Supporting information

S1 Fig Desirability function showing the predicted optimal factors for Pb removal. (TIF)

S1 Table GGDC analysis and comparison of MHSD_37 with reference genomes. (PDF)

S2 Table Multi-metal resistance of the *Bacillus* sp MHSD_37 strain based on positive from solid agar. (PDF)

S3 Table A summary of the plant growth promoting characteristic identified from the isolated strain *Bacillus* sp. MHSD_36. (PDF)
