# Supplementary figures and images for: Bioremediation of Pb contaminated water using a novel Bacillus sp. strain MHSD_36 isolated from Solanum nigrum
Source: PLoS One. 2024 Apr 29;19(4):e0302460. doi: 10.1371/journal.pone.0302460 (PMC11057764; doi:10.1371/journal.pone.0302460)

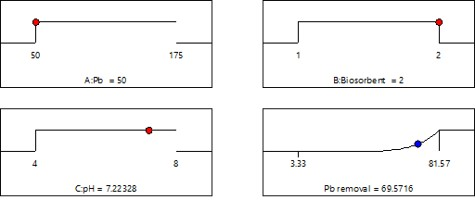

Supplement: S1 Fig — (TIF) [file pone.0302460.s001.tif]
