# Supplementary material for: Bioremediation of Pb contaminated water using a novel Bacillus sp. strain MHSD_36 isolated from Solanum nigrum
Source: PLoS One. 2024 Apr 29;19(4):e0302460. doi: 10.1371/journal.pone.0302460 (PMC11057764; doi:10.1371/journal.pone.0302460)
Supplement: S1 Table — (PDF) [file pone.0302460.s002.pdf]

		Formula 2				G+C difference
**Query genome**	**Reference genome**	**DDH**	**Model C.I.**	**Distance**	**Prob. DDH >= 70%**	
MHSD_36_2023	MACG00000000	53.6	[50.9 - 56.3%]	0.0638	30.13	0.1
MHSD_36_2023	MAOE00000000	58.2	[55.4 - 61%]	0.0548	46.21	0.36
MHSD_36_2023	MACE00000000	54.2	[51.5 - 56.9%]	0.0625	32.34	0.12
MHSD_36_2023	MACI00000000	43.7	[41.1 - 46.2%]	0.0881	6.27	0.14
MHSD_36_2023	MACD00000000	55.2	[52.5 - 57.9%]	0.0605	35.7	0.1
MHSD_36_2023	MACF00000000	53.6	[50.9 - 56.3%]	0.0637	30.34	0.01
MHSD_36_2023	AE016877	43.8	[41.2 - 46.3%]	0.0877	6.42	0.04
MHSD_36_2023	NC_022781	42.3	[39.8 - 44.9%]	0.092	4.71	0.29
MHSD_36_2023	LOBC01000053	95.8	[94.3 - 96.9%]	0.0057	97.38	15.76
MHSD_36_2023	NC_009674	25.4	[23.1 - 27.9%]	0.1709	0.01	0.56
MHSD_36_2023	BAUY00000000	38.3	[35.8 - 40.8%]	0.1054	1.73	0.11
MHSD_36_2023	MAOC00000000	40.1	[37.6 - 42.6%]	0.0992	2.77	0.01
MHSD_36_2023	NWUW00000000	52.7	[50 - 55.3%]	0.0657	27.06	0.33
MHSD_36_2023	ABJC00000000	52.2	[49.5 - 54.9%]	0.0667	25.6	0.15
