# Supplementary material for: Bioremediation of Pb contaminated water using a novel Bacillus sp. strain MHSD_36 isolated from Solanum nigrum
Source: PLoS One. 2024 Apr 29;19(4):e0302460. doi: 10.1371/journal.pone.0302460 (PMC11057764; doi:10.1371/journal.pone.0302460)
Supplement: S3 Table — (PDF) [file pone.0302460.s004.pdf]

Plant-growth-promoting characteristics	
Siderophore	+
IAA	+
ACC	+
Phosphate Solubilisation	+

+ Indicates the presence of a plant growth promoting characteristic if the isolated strain MHSD_36
